# Neutrophil dynamics and inflammaging in acute ischemic stroke: A transcriptomic review

**DOI:** 10.3389/fnagi.2022.1041333

**Published:** 2022-12-22

**Authors:** Truong An Bui, Glen C. Jickling, Ian R. Winship

**Affiliations:** ^1^Neuroscience and Mental Health Institute, University of Alberta, Edmonton, AB, Canada; ^2^Neuroscience and Mental Health Institute, University of Alberta, Edmonton, AB, Canada; ^3^Department of Medicine, Division of Neurology, Faculty of Medicine and Dentistry, University of Alberta, Edmonton, AB, Canada

**Keywords:** neutrophils, aging, inflammaging, stroke, ischemia, futile recanalization, collaterals, collateral circulation

## Abstract

Stroke is among the leading causes of death and disability worldwide. Restoring blood flow through recanalization is currently the only acute treatment for cerebral ischemia. Unfortunately, many patients that achieve a complete recanalization fail to regain functional independence. Recent studies indicate that activation of peripheral immune cells, particularly neutrophils, may contribute to microcirculatory failure and futile recanalization. Stroke primarily affects the elderly population, and mortality after endovascular therapies is associated with advanced age. Previous analyses of differential gene expression across injury status and age identify ischemic stroke as a complex age-related disease. It also suggests robust interactions between stroke injury, aging, and inflammation on a cellular and molecular level. Understanding such interactions is crucial in developing effective protective treatments. The global stroke burden will continue to increase with a rapidly aging human population. Unfortunately, the mechanisms of age-dependent vulnerability are poorly defined. In this review, we will discuss how neutrophil-specific gene expression patterns may contribute to poor treatment responses in stroke patients. We will also discuss age-related transcriptional changes that may contribute to poor clinical outcomes and greater susceptibility to cerebrovascular diseases.

## Cerebral ischemia and the ischemic cascade

Stroke is one of the leading causes of death and long-term disability globally (Sturm et al., 2004; Chen et al., 2010; Benjamin et al., 2019) and leads to significant healthcare and economic burden (Saka et al., 2009). A stroke can be characterized as ischemic or hemorrhagic (Donkor, 2018). Hemorrhagic stroke results from a ruptured blood vessel that bleeds into the surrounding area. Cerebral ischemia, or ischemic stroke (IS), is caused by a blockage of the artery supplying an area of the brain (Formisano et al., 2020). Over half of all IS occurs in the middle cerebral artery (MCA), one of the main blood supplies to the brain (Ng et al., 2007). The MCA derives directly from the internal carotid artery (ICA) and routes along the lateral sulcus before branching into the basal ganglia, frontal, parietal, and temporal lobes (Llovera et al., 2014). The MCA connects the anterior cerebral artery (ACA) and the posterior cerebral artery (PCA). Along with the ICA and PCA, these form the circle of Willis. There are four main branches in the MCA, termed M1, M2, M3, and M4. With respect to IS, the location of MCA occlusion determines the extent of cerebral injury. Proximal middle cerebral occlusion (MCAO) is an occlusion nearer to the origin of MCA and results in damage to a large cortical and subcortical region (Llovera et al., 2014). Distal MCAO is an occlusion more distally that damages a smaller area, often a purely cortical injury with a less severe clinical presentation (Bouet and Freret, 2012; Navarro-Orozco and Sánchez-Manso, 2021).

The pathophysiology of IS is complex. Focal IS begins with an occlusion of a cerebral artery (most commonly the MCA), leading to focal hypoperfusion in downstream territories. The lack of oxygen and reduced delivery of glucose in the ischemic region disrupts normal cellular ATP synthesis. This prompts cells to switch to anaerobic metabolism, promoting lactic acidosis and electrochemical gradient loss. Impaired ATP production prevents the normal function of ATP-reliant ion transport pumps, leading to cellular depolarization and intracellular Ca^2+^ elevation, which triggers free radical release, excitotoxicity, and cytotoxic edema (Dirnagl et al., 1999; Endres et al., 2008; Xing et al., 2012). Severe ischemia leads to rapid cell lysis due to anoxic depolarization, while less severe cellular injury triggers apoptosis. As the cell membrane and the mitochondria are damaged, there is an increase in intracellular toxins and apoptotic factors that initiates a caspase-dependent apoptotic cascade, causing cells to undergo cell death and further exacerbating local inflammation (Dirnagl et al., 1999; Allencherril et al., 2019). Without immediate restoration of blood flow, the ischemic cascade results in permanent cerebral damage (Hossmann, 2006; Durukan and Tatlisumak, 2007; Lakhan et al., 2009).

The rate and reversibility of IS vary with the degree of preserved blood flow and tissue viability at the time of treatment (Kalogeris et al., 2012). The current gold standard treatment for IS is recanalization through endovascular therapy (EVT), involving a catheter-based mechanical thrombectomy (MT), and adjunctive systemic intravenous (IV) thrombolysis with recombinant tissue plasminogen activator (r-tPA; Rothwell, 2018; Campbell et al., 2019). Both interventions aim to recanalize the affected blood vessels and reperfuse ischemic tissue to restore oxygen and nutrient delivery (del Zoppo et al., 1992; Panni et al., 2019). Studies have demonstrated the benefits of IV r-tPA for treating IS (Phan et al., 2017); however, its efficacy has been suboptimal, and many patients treated within its therapeutic window exhibited limited benefit (Allencherril et al., 2019). EVT is highly effective at opening large vessel occlusions and produces superior outcomes to IV thrombolysis (Goyal et al., 2016). Nevertheless, good outcomes are not always achieved with EVT, even when the vessel is fully recanalized. Clinical trials MR CLEAN, EXTEND-IA, ESCAPE, SWIFT PRIME, and REVASCAT showed the rate of “futile recanalization” after endovascular treatment to be 54%, even among patients with optimal conditions of recanalization timing and efficiency (Goyal et al., 2016; Leischner et al., 2019; Flottmann et al., 2021). These studies and trials suggest that tissue viability prior to recanalization is vital and that successfully reopening the vessels does not necessarily equate to improved tissue-level perfusion. This latter phenomenon of microcirculatory failure is known as “no-reflow.”

## Collateral circulation and stroke outcome

In the late 1970s, Lynsay Symon demonstrated that in reversible MCAO, there is the presence of the ischemic penumbra and oligaemia, in addition to an ischemic core where irreversible neuronal death occurs within minutes after the occlusion (Astrup et al., 1981; Baron et al., 2020). In this core/penumbra model, Symon showed that there are three parts in the ischemic area, (1) the ischemic core with established irreversible cell death and the cerebral blood flow below 10 ml/100 g/min (as to a normal 50 ml/100 g/min flow); (2) the ischemic penumbra outside of the core with reduced perfusion rate but viable and salvageable if blood flow is restored; and (3) the oligaemia that is mildly hypoperfused and neuronal functions are preserved (Astrup et al., 1981; Dirnagl et al., 1999; Jackman and Iadecola, 2015; Baron et al., 2020). Unless reperfusion occurs quickly, irreversible cell death in the core expands; hence, the famous mantra “time is brain.”

Collateral circulation refers to auxiliary vascular pathways that allow for partial perfusion of ischemic tissue after the primary vascular routes are blocked (Liebeskind, 2003, 2012; Shuaib et al., 2011; Winship, 2015). Collateral circulation consists of the primary and secondary arterial collaterals and venous collaterals. Primary arterial collaterals are the arterial segments in the circle of Willis that carry blood between the areas of the ICA and vertebrobasilar system or between cerebral hemispheres. The secondary collaterals include leptomeningeal arteries at the pial surface of the cortex, which connect the distal branches of the ACA, PCA, and MCA (Liebeskind, 2003; Shuaib et al., 2011; Winship et al., 2014; Winship, 2015). Under normal conditions, leptomeningeal collaterals are dormant, but are recruited when blood flow in major arteries is arrested. During MCAO, the change in pressure gradient between the areas supplied by the ACA or PCA permits blood to flow through leptomeningeal collateral anastomoses from intact areas into the ischemic region. Thus, the extent of collateral flow defines the viability of the penumbral region (Winship, 2015). Blood flow through these collaterals drops once the pressure gradient is restored after successful recanalization (Iwasawa et al., 2016).

Cerebral collaterals are associated with IS pathophysiology (Liebeskind, 2003), penumbral volume (Jung et al., 2013), and response to treatment (Becker, 1998). Collateral circulation deterioration after IS is also correlated with continued infarct growth in patients without recanalization (Campbell et al., 2013). Adequate collateral circulation, measured by collateral status, can temporarily maintain tissue viability in the absence of recanalization. Collateral status can be determined by using computed tomographic angiography and a variety of scales including the binary modified Tan collateral scale, in which “good collaterals” fill more than 50% of the area distal to MCAO region and “poor collaterals” can only achieve <50% (Tan et al., 2009; Yeo et al., 2015; Gensicke et al., 2022). In both IV thrombolysis and EVT trials, a shorter time to treatment correlated with better odds for positive outcomes (Lees et al., 2010; Khatri et al., 2014; Prabhakaran et al., 2015). However, many patients with good collateral flow and prolonged time from symptom onset to treatment of up to 24 h could still benefit from EVT (Albers et al., 2018; Nogueira et al., 2018). This shows that there is a patient-to-patient variation in collateral circulation, and that collaterals extend the time window to treatment and predict good clinical responses (Ribo et al., 2011; Hwang et al., 2015). Furthermore, patients with better angiographically assessed collateral scores have shown smaller infarct volumes, a lower degree of dependence and disability, and better functional recovery from IS (Becker, 1998; Christoforidis et al., 2005).

It has been shown that the prognosis of stroke patients is influenced by collateral circulation, especially leptomeningeal anastomoses. Collaterals sustain tissue viability until reperfusion (Tariq and Khatri, 2008). In recent years, evidence of leptomeningeal anastomoses contributing to ischemic tissue viability has been documented using computerized tomography (CT) angiography, triphasic perfusion CT, Xenon CT, and magnetic resonance imaging (Wildermuth et al., 1998; Brozici et al., 2003; Tariq and Khatri, 2008). In the PROACT II clinical trial, researchers showed that leptomeningeal collaterals are associated with better clinical outcomes (evaluated by NIHSS score), lower infarct size (shown in 24-h CT scan), and more rapid recanalization (Roberts et al., 2002). Similarly, other studies using CT showed that collaterals are correlated with lower infarct volume post-recanalization and better discharge mRS scores (Ringelstein et al., 1992; Christoforidis et al., 2005; Mohammad et al., 2008). Lee and their colleagues demonstrated using triphasic CT that slow or few meningeal collaterals corresponded to severe perfusion deficits and hemorrhagic transformation (Lee et al., 2000). In 2017, a retrospective study of 135 patients with ischemia and good collateral scores demonstrated that these scores significantly predicted successful recanalization, as determined by 90-day mRS scores (Tong et al., 2017). These findings provide concrete evidence of the correlation between good collaterals and improved recanalization outcomes post-stroke.

## Futile recanalization and the “no-reflow” phenomenon

The “no-reflow” phenomenon is defined as inadequate perfusion through a given segment of circulation without any angiographic evidence of mechanical vessel obstruction (Kloner et al., 2018; Allencherril et al., 2019; El Amki et al., 2020; Ter Schiphorst et al., 2021). This could be due to microcirculatory flow failure in small arterioles and capillaries despite a complete recanalization of the large, occluded artery (Soares et al., 2009; Pan et al., 2021). Although there are different processes involved in the ischemic cascade and IS pathogenesis, increasing evidence shows that inflammation is a primary driver of no-reflow (Muir et al., 2007; Jaffe et al., 2008; Kloner et al., 2018; Jayaraj et al., 2019).

The no-reflow phenomenon has been suggested to contribute to futile recanalization (El Amki and Wegener, 2017). Futile recanalization refers to the failure to improve neurological outcomes despite a successful recanalization, as indicated by the modified Rankin Scale (mRS) scores of 4–6 (Pan et al., 2021). The mRS measures the degree of disability or dependence in daily activities and is scaled from 0 (no symptoms) to 5 (severe disability) and 6 (death). Patients with near-complete or complete recanalization are divided into two groups, according to their functional outcomes at 3 months. Scores 0–2 are defined as “meaningful recanalization,” while scores 3–6 are “futile recanalization” (Patel et al., 2012; Saver et al., 2021). There are several factors that may account for futile recanalization, but neutrophil adhesion to the microvasculature has recently gained attention and may play a key role in no-reflow (Liebeskind, 2003; El Amki et al., 2020). Understanding the molecular changes in the hypoperfused tissues is paramount to resolving reperfusion injury, microcirculatory dysfunctions, no-reflow, and futile recanalization, as well as finding novel predictors of clinical deterioration and prevention strategies (Cowled and Fitridge, 2011). Notably, good collateral status is associated with reduced frequency of futile recanalization, as sufficient collateral flow may maintain tissue viability prior to restored flow in the occluded vessel (Bang et al., 2015). Moreover, good collaterals may also reduce the activation of neutrophils by cerebral ischemia, thereby reducing no-reflow/microcirculatory failure and improving tissue reperfusion after successful recanalization (Bang et al., 2015; Kloner et al., 2018).

## Post-ischemic inflammation exacerbates stroke outcomes

Depending on the injury severity, acute inflammation can initiate within minutes and resolve after days (Chung et al., 2019; Endres et al., 2022). In IS, acute inflammation manifests immediately after vessel occlusion (Iadecola and Anrather, 2011; De Meyer et al., 2016).

Following IS, the ensuing hypoxia, reduced sheer stress on the post-capillary venule walls, and reactive oxygen species (ROS) production trigger leukocytes recruitment, including neutrophils, lymphocytes, and monocytes, to the injury site (Dereski et al., 1993; del Zoppo, 1994; Iadecola and Anrather, 2011; Perez-de-Puig et al., 2015). These cells have long been documented in the peripheral blood samples of patients with IS (Huang et al., 2006; Whiteley et al., 2009; Kim et al., 2012; Petrovic-Djergovic et al., 2016; Xue et al., 2017). Cell recruitment prompts a systemic inflammatory reaction characterized by an activation of peripheral immune cells and the release of pro-inflammatory mediators from the ischemic endothelium and brain parenchyma (Iadecola and Anrather, 2011; Jickling et al., 2015; Planas, 2018). Inflammatory cells release several cytotoxic agents, such as matrix metalloproteinases (MMPs) and nitric oxide (NO). These mediators intensify cellular damage and disrupt the extracellular matrix and blood–brain barrier (BBB), and can lead to hemorrhagic transformation (Danton and Dietrich, 2003; Prass et al., 2003). Within 24 h after onset, cell damage, ATP, fibrinogen, and ROS activate microglia, the primary immune cells of the CNS (Weinstein et al., 2010; Anttila et al., 2017). Microglia produce inflammatory cytokines (e.g., IL-1β, IL-6, and TNF-α) and chemokines (e.g., MIP-1, MIP-2, and MCP-1) in the brain parenchyma (Raghavendra Rao et al., 2002), which stimulates NF-κB expression and the upregulation of adhesion molecules (E-selectin, L-selectin, P-selectin, ICAM-1, and integrins) on the endothelial cell surface (Morioka et al., 1993; Becker, 1998). Cytokine release also facilitates progressive cell death (Zhang et al., 1994) and oxidative cellular injury (Nagayama et al., 2000), exacerbating the ischemic cascade.

**Figure 1:**
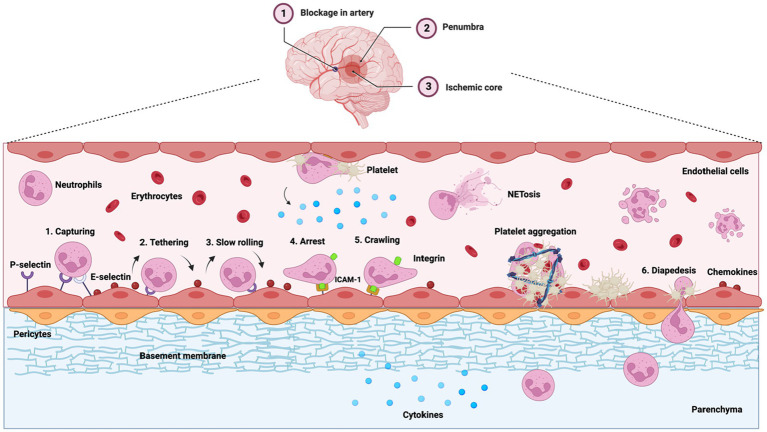
Leukocyte (neutrophil) extravasation and infiltration during inflammation are major contributors to poor ischemia outcomes. Middle cerebral artery occlusion is illustrated. During inflammation, cytokines (IL-1β, IL-6, and TNF-α) and the chemokines (MIP-1, MIP-2, and MCP-1) are upregulated, which stimulate the expression of selectins and ICAM-1 on the endothelial surface near the ischemic area. Chemokines (blue dots) are transcytosed through or deposited on the endothelium. E-selectin and P-selectin capture neutrophils from the blood flow, pulling the tethers at the rear of neutrophils and initiating their rolling motion. This activates integrins on neutrophils that firmly attach to ICAM-1, causing neutrophil adhesion. Neutrophils also recruit platelets, causing platelet aggregation and blocking the flow of red blood cells (erythrocytes). Neutrophils then infiltrate into the perivascular space through diapedesis while continuing to release pro-inflammatory factors into the ischemic area, causing neuronal damage.

Adhesion molecules permit leukocyte adhesion to the vascular endothelium and their infiltration into the brain parenchyma (Hallenbeck, 1996; Suzuki et al., 1997; Sughrue et al., 2004), which is a hallmark of acute inflammation (Perry and Granger, 1991; Bienvenu et al., 1992). Selectins generally slow down circulating leukocytes by attracting them to the endothelial surface. Upon activation, L-selectin recruits leukocytes to the injury site, while P-selectin and E-selectin bind to leukocytes on the endothelium (Becker, 1998), facilitating leukocyte rolling and adhesion (De Meyer et al., 2016; Petrovic-Djergovic et al., 2016; Figure 1). P-selection upregulation initiates as early as 15 min after ischemic onset while E-selection expression begins within 2 h (Yilmaz and Granger, 2008). Normally, leukocytes flow smoothly across capillaries without rotating or marginating. However, in IS, leukocytes, and specifically neutrophils, may clog the capillaries and disrupt blood flow (Doerschuk et al., 1993). Impeding inflammatory responses and, recently, reducing these neutrophil stalls have been shown to ameliorate injury in animal stroke models (Cuartero et al., 2013; Drieu et al., 2018; Kelly et al., 2018; El Amki et al., 2020; Erdener et al., 2021). For instance, inhibiting CXCR1 and CXCR2 in rat MCAO model, which are among the biomarkers of inflammation, improves behavioral measures, reduces ischemic brain damage, and decreases IL-1β level in the brain (Villa et al., 2007). IL1-receptor antagonist has also been found to decrease immune cell invasion in the brain, including one of neutrophils, as well as reducing infarct size and advancing behavioral outcomes in rodents (Rothwell, 2003). P-selectin expression is also associated with a reduced cerebral blood flow post-reperfusion, and animals that lack P-selectin have smaller infarct size and better survival (Connolly et al., 1997). ICAM-1 and integrins control leukocyte adhesion and movement across endothelial cells, or diapedesis, and eventual migration to the parenchyma (Blann et al., 2002). This can be observed as early as 30 min after onset and prolongs to the 48-h mark (Yilmaz and Granger, 2008). When leukocytes attached to adhesion molecules are activated, they produce and release ROS, proteases (e.g., elastase, collagenase, and gelatinase), and cationic proteins (e.g., defensins) that can proteolyze the endothelial membrane and interstitial matrix (Weiss, 1989; Granger, 1999). Blocking ICAM-1, thus reducing leukocyte adhesion, has been shown to reduce IS damage (Zhang et al., 1995).

Days and weeks after the initial insult, neutrophils and other inflammatory cells gradually undergo efferocytosis (a programmed cell death that is similar to apoptosis but engages a different signaling cascade), in which they are phagocytosed by macrophages to resolve inflammation (Schwartz and Baruch, 2014; Zhu et al., 2019; Cai et al., 2020; Sendama, 2020). Efferocytosis removes apoptotic neutrophils that could otherwise undergo necrosis and prevents further neutrophil recruitment (Huynh et al., 2002; Rydell-Törmänen et al., 2006). This dual role of inflammation in both injury and repair poses many unwanted side effects to anti-inflammatory treatments for IS, which is important to address, especially with current challenges in clinical translation despite promising preclinical results.

In recent years, multiple clinical trials have been conducted to determine whether anti-inflammatory strategies are beneficial to IS, including studies on anti-integrin (HALT stroke study, ASTIN), anti-ICAM-1 (Enlimomab Acute Stroke Trial), and minocycline (MINOS, MINOS-sub analysis, NeuMAST; Jiang et al., 1995; Becker, 1998; Caimi et al., 2001; Gendron et al., 2002; Lampl et al., 2007; Martin et al., 2008; Perez-de-Puig et al., 2015). Despite promising preclinical data, these trials failed to show benefits in patients with stroke. The rationale for these failures is multifactorial and includes challenges in both the preclinical evaluation and the clinical trials conducted (Jiang et al., 1995; Becker, 1998; DeGraba, 1998; Yenari et al., 1998; Perez-de-Puig et al., 2015; Lambertsen et al., 2019). For instance, some trials failed to account for species differences (Schneider et al., 1998; Perez-de-Puig et al., 2015) with regard to treatment responses (Kim et al., 2014; Jickling et al., 2015) and cerebrovascular collateralization (Del Zoppo, 1995; Liebeskind, 2003). The true goal of anti-inflammatory IS treatments should be inhibiting persistent detrimental inflammatory processes and restoring tissue homeostasis, rather than blocking all immunoregulatory functions (Lawrence and Gilroy, 2007).

## Neutrophil stalls in capillaries aggravate inflammation and hypoperfusion

During IS, the stroke core develops severe focal hypoperfusion and irreversible injury and becomes a nidus for inflammation. The penumbra, while being more salvageable, is still at risk from ongoing ischemia (Shekhar et al., 2018; Semerano et al., 2019). These injuries can be aggravated by secondary damage occurring days or weeks after the initial insult (Chamorro et al., 2016). In addition to poor collaterals and collateral failure, which contribute to viability before recanalization (Hill et al., 1999; Boutin et al., 2001; Iadecola and Alexander, 2001), secondary microcirculatory failure (e.g., neutrophil stalls) in ischemic and penumbral regions (Jickling et al., 2015; Anrather and Iadecola, 2016) contributes to reperfusion failure and futile recanalization (Dalkara and Arsava, 2012; Iwasawa et al., 2016; Sheth et al., 2016; Erdener et al., 2021).

### Neutrophils as an inflammatory modulator

Neutrophils, also known as polymorphonuclear leukocytes, are the most abundant white blood cell in humans, representing 50%–70% of leukocytes (Furze and Rankin, 2008; Perez-de-Puig et al., 2015; Nah et al., 2018). These cells are continually produced in the bone marrow through granulopoiesis and released into circulation, constituting the first line of defense in the innate immune response under homeostatic conditions (Furze and Rankin, 2008; Mayadas et al., 2014; Christoffersson and Phillipson, 2018). Neutrophils are short-lived, with an estimated cellular lifespan of ~10–18 h once released into the bloodstream, although they can survive longer (Pillay et al., 2010; Lahoz-Beneytez et al., 2016; Ballesteros et al., 2020).

Neutrophils are complex, transcriptionally active cells that perform their immune functions *via* phagocytosis, granules production, degranulation, neutrophil extracellular traps (NETs) release, and reactive species generation (Borregaard, 2010; Pillay et al., 2010; Kolaczkowska and Kubes, 2013; Lahoz-Beneytez et al., 2016; Sollberger et al., 2018; Ballesteros et al., 2020). During cellular differentiation in the bone marrow, neutrophils are densely packed with secretory and antimicrobial granules to be released upon encountering pathogens (Gullberg et al., 1997; Mitsios et al., 2007; Ericson et al., 2014). Neutrophils generate web-like structures known as NETs, which are extracellular fibers composed of DNA-histone complexes and antimicrobial granule proteins (Lacy, 2006; Masucci et al., 2020). They are highly effective in trapping and killing pathogens by releasing enzymes, histones, and DNA out of the cells before dying through a process called NETosis (Becker, 1998; Brinkmann and Zychlinsky, 2007). NETs-forming neutrophils are found throughout ischemic brain tissue (Laridan et al., 2017; Denorme et al., 2022).

**Figure 2:**
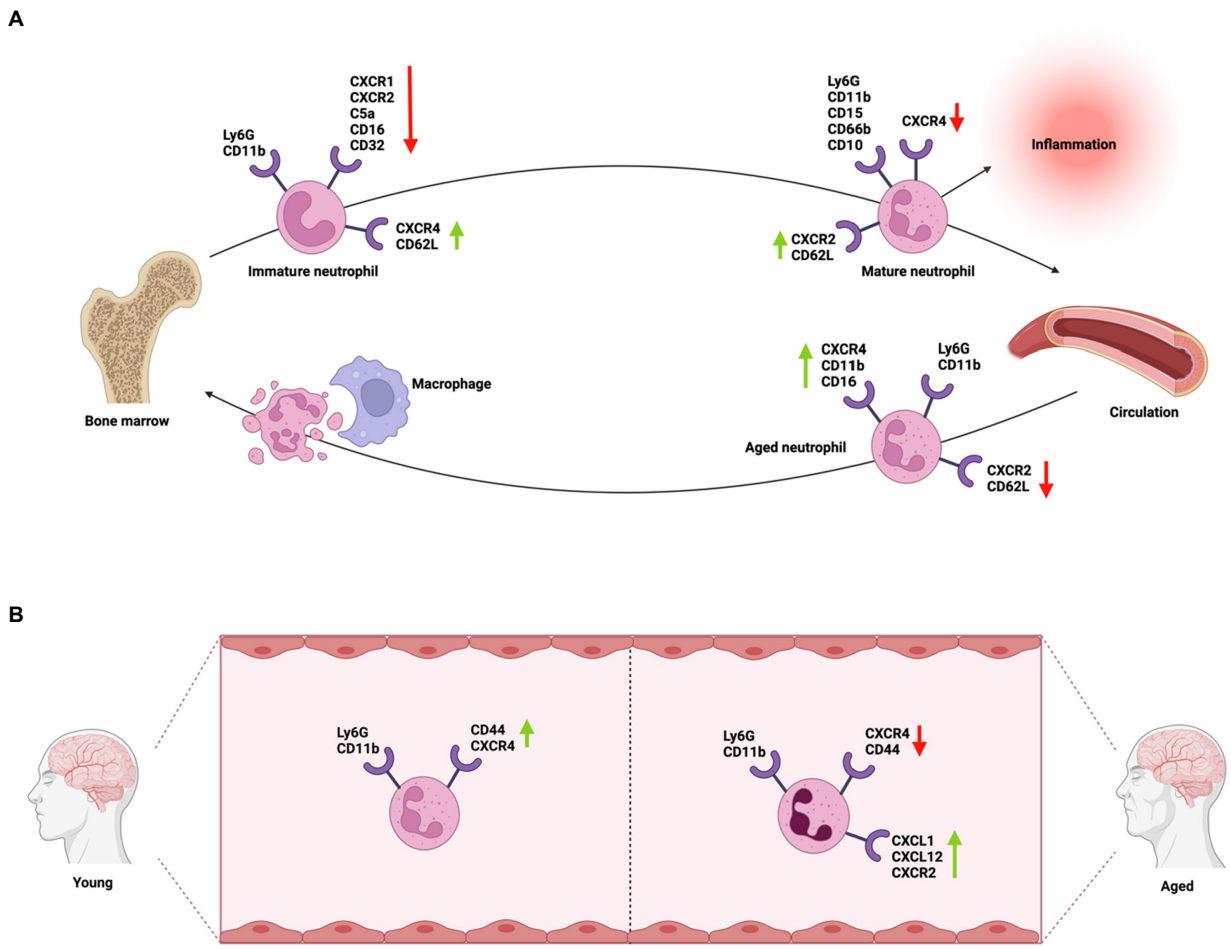
Neutrophil phenotypic differentiation during circulation and neutrophil phenotypic heterogeneity in aging. **(A)** Immature neutrophils are produced in the bone marrow, where they express either CXCR4 and CD62L on the cell surface to remain in the bone marrow or CXCR2 to be released into circulation as mature neutrophils. Aged neutrophils, after completing their immune functions in the circulation, have an increased expression of CXCR4 and a decreased expression of CXCR2 and CD62L to prompt their return to the bone marrow. **(B)** During organismal aging, circulating neutrophils demonstrated phenotypic changes compared to those in young individuals. Circulating neutrophils in aged individuals are hyperactive, having decreased CXCR4 and CD44 expression that diminish their efferocytosis and clearance. They also express high levels of CXCR1, CXCL1, and CXCL12 to help them remain in circulation, thus, creating a pro-inflammatory microenvironment. This condition of inflammaging makes aged individuals more susceptible to inflammatory stimuli.

As neutrophils circulate, they undergo “neutrophil aging,” a natural process that involves phenotypic and functional alterations that are distinct from organismal aging and follows a rigid diurnal regime (Martin et al., 2003; Casanova-Acebes et al., 2013; Zhang et al., 2015). This diurnal program of neutrophils is coordinated by CXCR2, which drives neutrophil aging, and CXCR4, which antagonizes it (Adrover et al., 2019). After their production, immature neutrophils are retained in the bone marrow through the ligation of CXCR4 receptor expressed on their surface and CXCL12. Immature neutrophils can be differentiated from the mature ones by their low-density fractions (Martin et al., 2003; Leliefeld et al., 2016; Figure 2). The upregulation of CXCR2 (ligands IL-8/CXCL1 and 2), along with G-CSF cleaving CXCR4-CXCL12 ligation *via* Cathepsin G and neutrophil elastase, release mature neutrophils from the bone marrow into circulation. However, these stimulators can also release immature neutrophils into circulation during significant inflammatory events (Lévesque et al., 2003; Grieshaber-Bouyer and Nigrovic, 2019). Mature neutrophils are morphologically smaller and can migrate to the injury site faster than the immature ones with enhanced phagocytic activities (Zhang et al., 2015; Uhl et al., 2016; Liu et al., 2021). As neutrophils circulate, they have increased CXCR4 and decreased CD62L expression, which triggers clearance by macrophages *via* efferocytosis to return to the bone marrow (Martin et al., 2003; Stark et al., 2005; Furze and Rankin, 2008; Casanova-Acebes et al., 2013; Figure 2). Thus, aged neutrophils have a shorter lifespan and faster apoptosis (Van Eeden et al., 1997; Simon, 2003; Milot and Filep, 2011; Casanova-Acebes et al., 2013; Drifte et al., 2013). These indicate that during inflammation, there are subsets of neutrophils in circulation with different functional properties (Perez-de-Puig et al., 2015).

A major explanation of neutrophil aging is to allow for temporal separation between subsequent neutrophil-mediated inflammatory responses, thus, honing their protective effects without damaging the vasculature (Adrover et al., 2019). Neutrophil aging has been correlated with various immunological and cardiovascular pathologies, including IS, atherosclerosis, sickle cell disease, rheumatoid arthritis, and asthma (Elliott, 1998; Coller, 2005; Scheiermann et al., 2013; Al-Ahwal et al., 2019; Esposito et al., 2020). In both humans and mice, neutrophil aging peaks in the morning, even though these two species have opposite circadian activities (Adrover et al., 2019). Consistent with neutrophils’ oscillatory signals and diurnal phenotypic changes, there is also a circadian variation in IS onset and progress (Aroca-Crevillén et al., 2020). Compared to hemorrhagic stroke and transient ischemic attack, IS has the highest risk between 6 a.m. and 12 p.m. but the lowest risk at night time between 12 a.m. and 6 a.m. (Elliott, 1998; Butt et al., 2009; Al-Ahwal et al., 2019). This may be partially explained by the rhythmic increase in immune activity and neutrophil infiltration (Scheiermann et al., 2013). Ischemic events that happen during sleep were found to have the worst prognosis and worst functional outcomes (measured by mRS), compared to those that occur during daytime (Jiménez-Conde et al., 2007; Al-Ahwal et al., 2019). In rodent models of stroke, it was shown that neuroprotective treatments can reduce infarct volume during their daytime onset (inactive phase), which corresponds to night time onset in humans, but not at the animals’ night onset (active phase; Esposito et al., 2020). Although inactive phase strokes are less frequent, they are severe with more cell death and infarct growth (Castellanos et al., 2008; Simonsen et al., 2016; Esposito et al., 2020). These patterns suggest an important pathological link between inflammation, neutrophils, and IS.

### Neutrophil functional dynamics post-ischemic stroke

In IS, the same functions that make neutrophils an effective defense against pathogens also cause a significant amount of bystander tissue damage (Furze and Rankin, 2008; Mayadas et al., 2014), as documented in human patients (Jickling et al., 2015; Perez-de-Puig et al., 2015). Neutrophils are among the first responders to the ischemic brain (Gelderblom et al., 2009; Wu et al., 2015). Their increased cell counts in circulation are observed as early as 4–6 h after IS (Wang et al., 2007), and their migration into the brain parenchyma begins after 6–8 h (Jickling et al., 2015). NETs are detected 2–3 days after stroke (Cai et al., 2020). Neutrophils continue to circulate as post-ischemic injury mounts, peaking at 1–3 days (Xu and Jiang, 2014) with expression still being detected after 7–15 days, then declining afterwards (Becker, 1998).

Neutrophil influx post-ischemia could lead to microcirculatory failure due to microvascular plugging, elevated blood viscosity, and increased vascular resistance (Engler et al., 1983; del Zoppo et al., 1991; Pham and Bendszus, 2016). While cytokines and vasoactive factors released by neutrophils might contribute to collateral failure, the consequences of these factors or a direct effect of neutrophil plugging on collateral flow have not been demonstrated. As previously discussed, during IS, endothelial adhesion molecules are rapidly upregulated, expressing integrins (e.g., α2β1, α4β1, and α6β1), recognition molecules (e.g., chemokine receptors CCR1, CCR2, CCR3, CCR5, CXCR3, and CXCR4), and proteases (Nourshargh and Marelli-Berg, 2005; Hartl et al., 2008). These molecules recruit neutrophils *via* the chemotaxis thrombin (Reglero-Real et al., 2016). Thrombin can prolong neutrophil-adhesion molecules interactions by activating protease-activated receptors and NF-κB. It can also directly upregulate both C3 and C5 components of the complement system to disrupt endothelial barrier functions (Becker, 1998; Xue et al., 2009). These promote neutrophil occlusion in the capillary bed in mostly the stroke core and the penumbra (Jiang et al., 2014). Neutrophil occlusion is a prime contributor to tissue loss and unfavorable outcomes in patients receiving thrombolysis (Malhotra et al., 2018). Neutrophil adhesion to the endothelium could also lead to accumulated stalls and block erythrocyte movement through the microvasculature, resulting in infarct expansion (Rolfes et al., 2021). Higher neutrophil counts and infiltration are associated with larger infarct volumes post-IS and elevated neutrophil counts at admission are also associated with poor clinical outcomes (Price et al., 2004; Semerano et al., 2019). Thus, neutrophil depletion can reduce overall tissue damage, including infarct volume, edema formation, and hemorrhagic transformation in various animal stroke models, including MCAO mice and rats (Harris et al., 2005; Villa et al., 2007; Kenne et al., 2012; Cuartero et al., 2013; Herz et al., 2015; Wang et al., 2019; El Amki et al., 2020; Kang et al., 2020; Erdener et al., 2021). Blocking neutrophil entry into the brain can also drastically improve neurological outcomes in both MCAO rats and mice, evident in improved neurodeficit scores (e.g., forelimb strength, weight bearing, barrel rolling), rotarod, tight rope, corner turn, hanging wire, adhesive removal tests, T maze, foot-fault, water maze, and Garcia tests (Doycheva et al., 2014; Neumann et al., 2015; Zhao X. et al., 2017; Wang et al., 2019; El Amki et al., 2020; Roy-O’Reilly et al., 2020).

Aggregated neutrophils in capillaries have extended circulating time and increased cytotoxicity (Hartl et al., 2008; Allen et al., 2012; Dixon et al., 2012). Activated neutrophils at the surface of the endothelium release pro-inflammatory factors in and around the penumbra such as MMPs, ROS, and NOS. These destabilize the BBB, destroy the surrounding vasculature, and inflict secondary tissue damages (Garcia et al., 1994; Becker, 1998; Forster et al., 1999; Rosell et al., 2008; Yilmaz and Granger, 2008; Ludewig et al., 2013; Garcia-Bonilla et al., 2014; Perez-de-Puig et al., 2015; Figure 1). Neutrophil overproduction of MMPs can also lead to hemorrhagic transformation (Perez-de-Puig et al., 2015). Phospholipase activation in neutrophils produces prostaglandins and platelet-activating factors (Garcia et al., 1994), causing platelet aggregation, vasoconstriction, and flow stagnation (del Zoppo et al., 1991; Dawson et al., 1996; Ramiro et al., 2018). Neutrophils can then infiltrate the parenchyma and accumulate, inducing further neutrophil recruitment by continually releasing additional factors (e.g., ROS, cytokines, and proteases; Lorant et al., 1995; Wang et al., 2008; Tokgoz et al., 2013; Figure 1).

In addition to neutrophil stalls, NETs play a significant role in the no-reflow phenomenon. NETs close the time window for thrombolytic therapy and cause tPA resistance by promoting secondary thrombosis (Perez-de-Puig et al., 2015). NETs can also facilitate fibrin deposition, form scaffolds enclosing platelets, and activate the intrinsic coagulation pathway (Fuchs et al., 2010; Ionita et al., 2010; Massberg et al., 2010; Perez-de-Puig et al., 2015; Sørensen and Borregaard, 2016). NETs increase inflammatory cytokine levels and trigger the pro-inflammatory microglia subtype (Perez-de-Puig et al., 2015; Hanhai et al., 2021). The frequency and intensity of NETosis are significantly greater after permanent MCAO, and NETs marker levels are linked to stroke severity in patients, as evaluated by NIHSS and mRS scores (Vallés et al., 2017).

**Figure 3:**
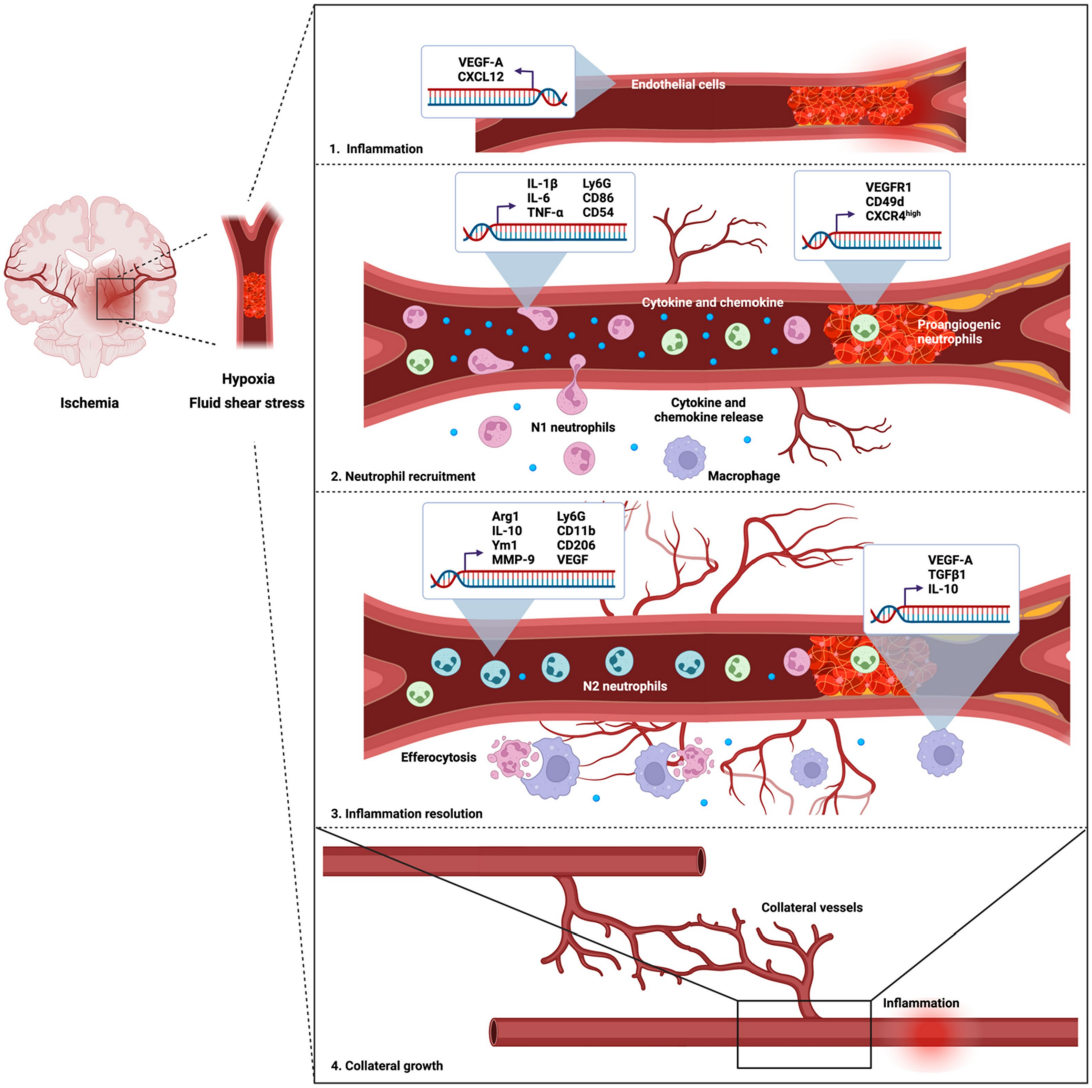
Different subpopulations of neutrophils have distinct effects on collateral growth and angiogenesis post-ischemia. Angiogenesis post-ischemia stimulates the growth of new collateral vessel conduits, which helps partially perfuse the tissues and sustain tissue viability until recanalization and reperfusion. Middle cerebral artery occlusion is depicted with collateral vessel growth post-ischemia. Three subtypes of neutrophils, pro-inflammatory N1, protective N2, and pro-angiogenic neutrophils are recruited to the inflammatory site post-IS in response to hypoxic stimuli and fluid shear stress. During ischemic injury, hypoxic genes, including VEGF-A, CXCL12, and DAMPs are upregulated. VEGF-A is overly expressed in endothelial cells, which recruit N1 neutrophils and pro-angiogenic neutrophils into the circulation. The later wave of recruitment consists of both pro-angiogenic and N2 neutrophils that initiate collateral development via MMP-9 release. This prompts collaterals recruitment from adjacent vascular arteries. Resolution of inflammation is completed by efferocytosis of neutrophils by macrophages.

Neutrophils differ in their expression of surface markers and nucleus density (Perez-de-Puig et al., 2015). This heterogeneity impacts their immune functions (Perez-de-Puig et al., 2015). Ischemic environments and neutrophil interaction with endothelial adhesion molecules shift neutrophil phenotype from the protective N2 to the injurious N1 phenotype (Nourshargh and Marelli-Berg, 2005; Perez-de-Puig et al., 2015). N1 neutrophils have hyper-segmented nuclei that are seen in mature neutrophils while N2 neutrophils have banded or ring-shaped nuclei exhibited in immature neutrophils (Eruslanov et al., 2017). N1 neutrophils secrete inflammatory molecules, such as cytokines and effector molecules, including IL-1β, IL-12, TNFα, IFNγ, NO, CXCL13, CCL3, CC6, CXCL10, and hydrogen peroxide (Andzinski et al., 2016; Sionov, 2021). N1 neutrophils are also characterized by the surface expression of Ly6G, CD11b, CD54, and CD86 (Wang et al., 2018; Li et al., 2019; Figure 3). These cells are short-lived, highly cytotoxic and can worsen inflammatory damage. In contrast, N2 neutrophils are long-lived and anti-inflammatory. These cells express Ly6G, CD11b, CD206, MMP9, ARG1, and YM-1; and emerge at later stages (days 5–7; Rao et al., 2014; Perez-de-Puig et al., 2015; Ma et al., 2016; Figure 3). N2 cells produce anti-inflammatory cytokines, including TGFβ, IL-10, CCL2, CCL5, CCL17, CXCL4, and VEGF, which confer neuroprotection, tissue remodelling, and wound healing (Shirasuna et al., 2013; Perez-de-Puig et al., 2015; Sionov, 2021). It is notable here that VEGF upregulation confers biphasic roles in IS, with both deleterious impacts such as BBB disintegration and hemorrhagic transformation within 24 h and angiogenic functions 48 h post-ischemic onset (Zhang et al., 2000; Suzuki et al., 2016; Hu et al., 2022). N2 neutrophils facilitate neutrophil clearance by efferocytosis and are less harmful to ischemic neurons (Cuartero et al., 2013; Christoffersson and Phillipson, 2018; Cai et al., 2020). Skewing neutrophils toward the N2 phenotype with TGFβ treatment before IS has been shown to significantly reduce infarct volumes in MCAO mice (Cai et al., 2020). The binary classification of N1 and N2 neutrophils, in recent years, has been suggested to be oversimplified, as neutrophil phenotypes can exist as a continuum of activation states instead of extreme dichotomy (Eruslanov et al., 2017; Jaillon et al., 2020). One example of this is the recently discovered pro-angiogenic neutrophil subtype, which constitutes ~3%–5% of circulating neutrophils in both humans and mice. They have angiogenic properties (Tsuda et al., 2004; Fridlender et al., 2009; Christoffersson and Phillipson, 2018) and may contribute to collateral growth post-IS by releasing growth factors and pro-angiogenic factors (Jayaraj et al., 2019; Phillipson and Kubes, 2019). This type of neutrophils is prone to hypoxic stimulus and is recruited by increased VEGF-A expression, a potent inducer of endothelial cell chemotaxis (Barkefors et al., 2008; Piccard et al., 2012). They express high levels of CD11b, CXCR4, and VEGFR1 (Christoffersson et al., 2010, 2012; Massena et al., 2015) and overly express MMP-9, an angiogenic effector protein (Figure 3). MMP-9 can degrade the extracellular matrix to stimulate revascularization and neoformation of immature vessels at the ischemic site (Christoffersson et al., 2012; Massena et al., 2015). Pro-angiogenic neutrophils also engage different adhesion molecules, such as integrin VLA-4, in which its inhibition reduces new vessel growth and pro-angiogenic neutrophil presence at hypoxic site (Massena et al., 2015). Thus, these neutrophils increase the number of new vessels formed, including collateral conduits that can enhance blood supply during recovery (Weisenburger-Lile et al., 2019). Moreover, because collaterals reduce acute ischemia and maintain tissue viability, they might also reduce the activation of neutrophils via this reduced ischemia. Since distinct subpopulations of neutrophils perform opposing roles, targeting all neutrophils may not be ideal in stroke. A complete neutrophil removal, although showed positive effects in animal studies (Cuartero et al., 2013; El Amki et al., 2020; Erdener et al., 2021), is an impractical treatment clinically, as it requires an extensive amount of time and needs to be done before the stroke (Erdener et al., 2021). Complete inhibition of neutrophils could also increase risk of infection, as seen in patients with leukocyte adhesion deficiency (Perez-de-Puig et al., 2015). While the mechanism for therapeutic strategies for IS gearing against neutrophil adhesion is unclear and requires further investigation, it remains a promising avenue of research.

### Neutrophils, collaterals, and microvascular circulation

In addition to the adverse effects of neutrophil stalls post-ischemia, neutrophil counts and density in blood at admission are linked to hemorrhagic complications in patients who received MT, long-term outcomes, and collateral growth (Easton, 2013; Tokgoz et al., 2013, 2014; Xue et al., 2017). This is because continuous basal hyperactivation of circulating neutrophils can offset the equilibrium between apoptotic and necrotic immune cells, which may aggravate ischemic inflammation later (Perez-de-Puig et al., 2015). The extent of collateral circulation and the degree of reperfusion strengthen this association by modulating neutrophils’ capacity to reach the injury site (Semerano et al., 2019).

Neutrophil/lymphocyte ratio (NLR) has been proposed as a prognostic marker for inflammatory responses and clinical outcomes independent of age and recanalization (Brooks et al., 2014; Perez-de-Puig et al., 2015; Shantsila and Lip, 2015; Uysal et al., 2015; Albers et al., 2018; Giede-Jeppe et al., 2019; Li et al., 2021; Sharma et al., 2021). Prior to IS, neutrophils create a pro-inflammatory environment that can increase the severity of initial IS and further poor long-term outcomes (Kim et al., 2012; Zhang et al., 2020) while lymphocytes could induce neuroprotection (Wheeler et al., 2004; Gillum et al., 2005; Horne et al., 2005; Macrez et al., 2011). Elevated admission NLR was connected to hemorrhagic complications in patients receiving MT (Easton, 2013; Tokgoz et al., 2013, 2014; Perez-de-Puig et al., 2015; Guo et al., 2016; Xue et al., 2017) and can predict 3-month mortality (Goyal et al., 2018). The NLR value is also significantly higher in severe stroke cases compared to mild and moderate strokes (Ying et al., 2021).

With their different subpopulations, N1, N2, and proangiogenic neutrophils could exert both deleterious and protective effects on collaterals recruitment post-ischemia. During IS, peripheral neutrophils infiltration into the ischemic territory via circulation is severely reduced and their access is provided mainly through collateral blood vessels, peripheral brain regions, or when the ischemic areas are reperfused (Zhang et al., 1995; Prestigiacomo et al., 1999; Cuartero et al., 2013; Chu et al., 2014; Perez-de-Puig et al., 2015; Otxoa-de-Amezaga et al., 2019). Without reperfusion, the main route for neutrophil infiltration and extravasation is via retrograde collateral pathways, moving from leptomeningeal vessels to perivascular space and then parenchyma (Perez-de-Puig et al., 2015; Otxoa-de-Amezaga et al., 2019). Through this route, neutrophils and their NETs impair revascularization and tissue integrity (Yipp and Kubes, 2013) and patients with NETs-poor thrombi have better collateral flow and milder clinical symptoms with reperfusion (Kang et al., 2020; Chen et al., 2022). This suggests that treatments targeting NETs in thrombi might be beneficial for early neurological protection (Kang et al., 2020). Neutrophil influx and high local neutrophil density in circulation (counts/μL) are also correlated with poor retrograde collateral flow and greater infarct extent (Strinitz et al., 2021). Even in the subgroup with successful reperfusion and good collaterals, high neutrophils and low lymphocytes (thus, a higher NLR) are indicative of intracerebral hemorrhage (Guo et al., 2016; Gill et al., 2018; Semerano et al., 2019), collectively due to the destructive effects of neutrophils and the lack of vessel protecting effects from lymphocytes (Salas-Perdomo et al., 2018; Sharma et al., 2021, 2022).

There is significant evidence suggesting that ischemia, more specifically ischemic hypoxia and fluid shear stress, stimulates collateral circulation development in the peri-infarct regions after injury, as a result of angiogenesis and arteriogenesis (Wei et al., 2001; Heil et al., 2006; Faber et al., 2014; Phillipson and Kubes, 2019). Both angiogenesis and arteriogenesis presence after stroke have been observed in various animal models (Kanazawa et al., 2019). In rodents and non-human primates, angiogenesis can be detected as early as 4 days after ischemic onset in the peri-infarct region (Ijichi et al., 1995; Abumiya et al., 1999; Zhang et al., 2000; Tagaya et al., 2001; Hayashi et al., 2003; Wang et al., 2009; Li et al., 2012). An argument in favor of this is the upregulation of VEGF-A and EPO, which are central to collateral sprouting, at hypoxic sites (Semenza, 1999; Matsunaga et al., 2000; Resnick et al., 2003; Toyota et al., 2004; Kanazawa et al., 2019). VEGF recruits proangiogenic neutrophils selectively (Eltzschig and Carmeliet, 2011; Massena et al., 2015), which then release MMP-9 that breaks down the extracellular matrix, allowing for vessel growth by 4–7 days after focal ischemia (Grunewald et al., 2006; Nozawa et al., 2006; Christoffersson et al., 2012; Sun et al., 2017; Figure 3). VEGF-A has been implicated in collateral development (elevated pial collateral count), reduced infarct expansion after MCAO, and increased recanalization of occluded arteries (Clayton et al., 2008; Greenberg and Jin, 2013; Perez-de-Puig et al., 2015). Additionally, upon VEGF administration, both rat and mouse models have been shown to have higher new vessel density and better functional recovery (Zhang et al., 2000; Hayashi et al., 2003).

Another stimulator of post-ischemic collateral development is the fluid shear stress. The change in vascular tone and pressure during IS upregulates adhesion molecules expression, such as selectins, I-CAM, and V-CAM along the lumenal surface of collateral vessels (Scholz et al., 2000; Lee et al., 2004). Adhesion molecules recruit neutrophils to the site to activate collateral remodelling (Hoefer et al., 2004) and their inflammatory effects sustain vessel growth (Becker, 1998; Okyere et al., 2020). Removing these adhesion molecules has been shown to attenuate arteriogenesis (Hoefer et al., 2004).

Neutrophils, baseline collateral status, collateral growth post-stroke, and stroke outcomes are inextricably connected. Neutrophil infiltration and the expression of their deleterious subset in collateral vessels during IS can exacerbate stroke pathogenicity despite good collateral status at admission. On the other hand, the expression of pro-angiogenic and N2 neutrophils post-ischemia can enhance collateral growth during recovery. Although factors that determine stroke outcomes such as collateral status and circulating neutrophil levels at admission are fixed, many can still be done to improve functional recovery via post-ischemic neutrophil dynamics and collateral development manipulation (Wufuer et al., 2018; Wang et al., 2021). In patients with poor existing collateral networks, it may be possible to provide bone marrow-derived cell therapy (e.g., leukocytes and progenitor cells) to prompt endothelial functions and stimulate collateral growth and remodelling (Benest et al., 2008; Kinnaird et al., 2008). Upregulating pro-arteriogenic genes, such as VEGF, FGF, BDNF, and PDGF, after IS could also facilitate collateral development and functional recovery (Deindl et al., 2003; Clayton et al., 2008; Tomanek et al., 2008; Ergul et al., 2012; Chapouly et al., 2019).

## Neutrophil-specific transcriptional changes post-ischemic stroke

Altered genomic patterns have been observed in human blood samples as early as 3 h after an ischemic onset (Tang et al., 2006). In human brain tissue, many differentially expressed genes were found 2–6 days and 9–20 days after IS, with the majority of differentially expressed genes being upregulated. The number of upregulated genes in the rat brain tissues increases steadily after MCAO at 1 h, peaking at 3 days and persisting for several days, implying that active mechanisms are initiated during the acute phase of IS. Downregulated genes are detected 24 h after MCAO and their number declines after 26–37 days, indicating that gene expression is the most dynamic and robust during the first few weeks following stroke (Rusanen et al., 2015; Nannoni et al., 2019; Tarkanyi et al., 2020).

Transcription factors that lead to inflammatory responses, necrosis in the anoxic core, and delayed apoptosis in the penumbra have been documented (Zhan et al., 2011; Drifte et al., 2013). For instance, although has been recognized as a passive process lacking genetic regulation, a recent study in *Drosophila* and MCAO rats showed that necrosis is regulated by MSK1/2 and JIL-1 through H3S28ph phosphorylation (Liu et al., 2014). Numerous studies were done on apoptosis gene expression in the penumbra, highlighting the overexpression of Smac/DIABLO, AIF, XAF1, HtrA2/Omi, CASP3, etc. (Uzdensky, 2019). Changes in gene expression of circulating peripheral immune cells have also been recorded, including neutrophils, platelets, and cytokines (Lee et al., 2004; Meisner and Price, 2010; Zbinden et al., 2010). However, relatively few studies have been conducted on the transcriptional regulatory effects of IS on neutrophils alone (Konstantinov et al., 2004). Using microarray analysis, Tang et al., 2006 identified a total of 29 probe sets with 18 signature genes that distinguish IS from healthy human controls, with most highly expressed by neutrophils, suggesting that they are among the molecular and genomic signatures of stroke (Tang et al., 2006). Similar results were obtained by Carmona-Mora et al. using RNA-seq analyses of human blood samples. They found that the majority of the differentially expressed genes (197/248, 79%) were upregulated in IS neutrophils (Carmona-Mora et al., 2021). This reinforces the concept that post-stroke transcriptional changes occur predominantly in neutrophils (Moore et al., 2005). Some signature neutrophil genomic markers include FPR1 and PGLYRP1, which represent the initial steps leading to neutrophil activation and granulocyte accumulation at the inflammation site (Le et al., 2002; Tang et al., 2006); S100P/A8/A9/A12 for binding specifically to endothelial cells and induce inflammatory response; and NCF-4 that produces free radicals in neutrophils (Tang et al., 2006; Perez-de-Puig et al., 2015). Immediately after IS, mRNA of neutrophil chemoattractant (e.g., CXCL1, CXCL2, CXCL3, CCL5) are expressed, which peaks after 12 h and returns to the sham level at 48 h. After a day, IL-12, MMP3, TIMP1, MPO, and NE expression associated with neutrophil extravasation is increased. The surge in the expression of MMP10, NLRP3, and BAFF is not detectable until after 2 days, while IL-23, IFNγ, MMP2, MMP8, MMP9, MMP13, PAD4, TLR7, and TLR9 not until the third day. In the protective N2 neutrophils only, CD206 is upregulated after 7 days post-MCAO (Cai et al., 2020).

Neutrophils activate many canonical pathways implicated in IS. Very few neutrophil-specific pathways are suppressed post-ischemic stroke (Carmona-Mora et al., 2021). Significant and well-studied neutrophil-specific pathways post-ischemia include mTOR (Maiese, 2014), ERK5 (Becker, 1998), integrin (Bouchon et al., 2000), thrombopoietin (Yang et al., 2019), TGF-β, BMP, GM-CSF, IL-3, TRK, STAT3, and calpain protease signaling pathways (Carmona-Mora et al., 2021).

To exert its pro-death effects, neutrophils activate oxidative phosphorylation via cytokine signaling, calpain protease (Carmona-Mora et al., 2021), and thrombopoietin pathways (Bretón and Rodríguez, 2012). mTOR is activated via Akt and mediates pro-inflammatory gene transcription, autophagy, and apoptosis (Maiese, 2014; Hadley et al., 2019). PDGF, along with integrin signaling, stimulates chemotaxis and membrane ruffling before neutrophil extravasation (Perez-de-Puig et al., 2015; Lee and Li, 2018; Edwards and Bix, 2019). Integrins interact with extracellular matrix components to disrupt BBB permeability after stroke (Edwards and Bix, 2019). Calpain proteases further dysregulate synapses (Curcio et al., 2016) and cause BBB breakdown (Perez-de-Puig et al., 2015).

In parallel with pro-apoptotic pathways, neutrophils activate various neuroprotective pathways. ERK5 signaling, a MAP kinase family member, induces PPARδ, which is protective against stroke-induce brain injuries and its agonists are suggested as potential stroke treatments (Woo et al., 2006; Yin et al., 2011; Jin et al., 2013; Su et al., 2014). Neutrophin/TRK signaling activates hematopoietic cell survival and neurite outgrowth and differentiation (Reichardt, 2006). GM-CSF signaling, enriched at 48 h after stroke, initiates cell survival and differentiation (Cox et al., 1992; Becker, 1998; Lanfranconi et al., 2011; Carmona-Mora et al., 2021, 2022). Activation of TGF-β signaling, an N2 marker, exerts both deleterious and neuroprotective post-stroke, including upregulating the anti-inflammatory and anti-apoptosis IL-10 and IL-2 pathways (Boey et al., 1989, 1; Perez-de-Puig et al., 2015; Carmona-Mora et al., 2022). BMP signaling mediates glial scar formation after stroke (Shin et al., 2012) and TGFB3 signaling regulates N1 to N2 neutrophil polarization involved in inflammation resolution (Cuartero et al., 2013; Liao et al., 2014; Perez-de-Puig et al., 2015). These signaling pathways, once again, highlight the dual role of neutrophils during IS.

## “Inflammaging”

Organismal aging is characterized by a systemic dysfunction of the immune system and failed somatic maintenance, which involves mechanisms that maintain tissue integrity and promoted lifespan expansion. This leads to a chronic, sustaining inflammatory state (Kirkwood, 2005; Shaw et al., 2013; Childs et al., 2015; Perez-de-Puig et al., 2015; Boe et al., 2017; Rozhok and DeGregori, 2019). This phenomenon is referred to as “inflammaging” (Lencel and Magne, 2011; Franceschi and Campisi, 2014; Franceschi et al., 2018; Lu et al., 2022). 90% of the differentially expressed genes in human aged hippocampal brain tissue are linked to inflammation (Nikas, 2013). In a microarray study, aged hippocampal tissues showed an overexpression of pro-inflammatory genes, including (1) IL1B, IL6, IL10, and TNF that express cytokines, (2) IRAK3 and SOCS3 that modulate cytokine signaling, and (3) MYD88, TLR2, TLR4, and TLR7 that control TLR signaling (Cribbs et al., 2012). Inflammaging can diminish the immune system’s ability to fight and clear pathogens, causing greater susceptibility to infections, mortality, and age-related pathologies (Solana et al., 2006; Dorshkind et al., 2009; Lencel and Magne, 2011; Shaw et al., 2013; Pawelec et al., 2014; Akbar and Gilroy, 2020). The overall decline in immunity state is termed “immunosenescence” and is attributed to telomere erosion, defective protein catabolism, autophagy, and mitophagy (Perez-de-Puig et al., 2015).

**Figure 4:**
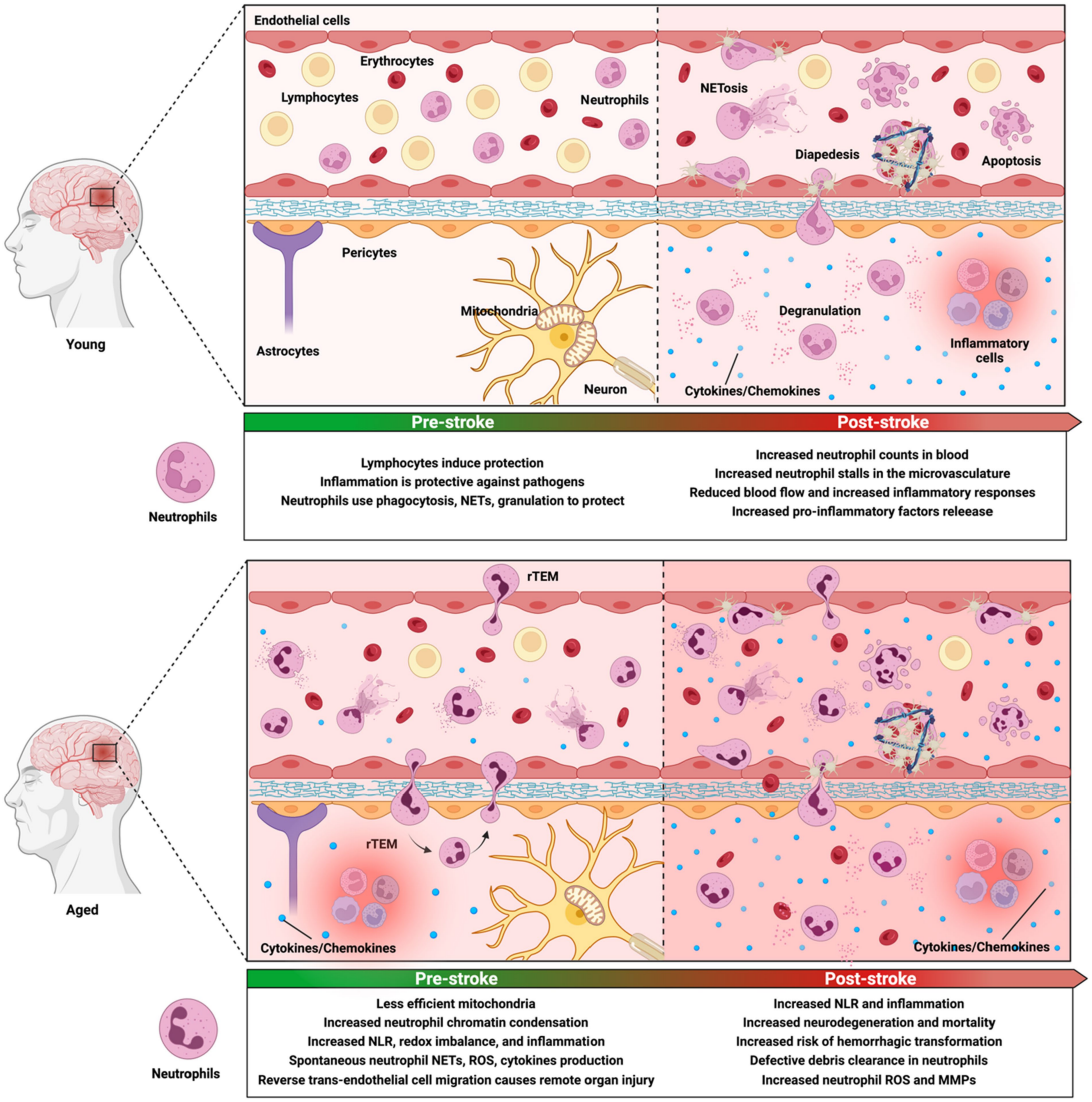
Aging alters the immune microenvironment as well as neutrophil phenotype and functions both before and after ischemic stroke. In normal condition, inflammation serves as a mechanism that protects the body against pathogens. Upon encountering pathogens, neutrophils undergo immune responses by producing granules, releasing neutrophil extracellular traps (NETs) and reactive species, and performing phagocytosis. Ischemic injury induces significant changes in the microvasculature, including increased neutrophil count, neutrophil stalls, and aberrant neutrophil activities that are correlated with neurological deficits and poor outcomes. During aging, the brain is primed with a basal elevated neuroinflammatory environment, which is evident in less efficient mitochondria, chemical gradient loss, and increased basal neutrophil counts and pro-inflammatory activities. With aging, due to increased levels of CXCL1 production, a higher level of neutrophil reverse transendothelial migration (rTEM) is seen. After neutrophils undergo diapesis, they can re-enter the vascular lumen and return to the circulation. They can stay in downstream organs, causing multiorgan failure. Aged individuals are significantly more susceptible to ischemic injuries than young individuals. They have increased risk of hemorrhagic transformation and worse neurological deficits. Their system has defective neutrophil clearance and heightened reactive species and MMP-9, causing sustained inflammatory responses. These further ischemic injuries and cause significantly more irreversible damage in aged subjects.

With age, the mitochondria slowly become less efficient (Sun et al., 2013; West et al., 2015), which compromises energy production, causing a redox imbalance (Harman, 1956). This promotes an overproduction of ROS, DAMPs (e.g., HMGN1 that regulates neutrophil activity), cardiolipin, and mitDNA that induces oxidative stress and inflammasome activation (Nakahira et al., 2015; Gkikas et al., 2018). Aging is also associated with an overall decline in the body’s anti-oxidative defense system, including efferocytosis (Sendama, 2020), which is supposed to remove cellular debris, senescent cells, (Xuan et al., 2011; Mohan et al., 2016; Wiggins and Clarke, 2019), and misfolded molecules, such as DNA, proteins, and lipids (Harman, 1956; Sohal and Orr, 2012). These molecules increase microglial pro-inflammatory cytokines, such as IL-6, TNF-α, IL-β, and microglial exposure to the anti-inflammatory TGFβ (Flanary et al., 2007; Perry and Holmes, 2014; Heneka et al., 2015; Figure 4). Chronic exposure of microglia to TGFβ impairs their capacity to secrete anti-inflammatory cytokines (Doyle et al., 2010; Cohen et al., 2014), producing more pro-inflammatory blood-borne factors and inflammasome (Villeda et al., 2011; Pishel et al., 2012; Perry and Holmes, 2014; Smith et al., 2015) and leading to chronic inflammation (Becker, 1998; Bruunsgaard et al., 2003; Ferrucci et al., 2005; Pinti et al., 2014). In many studies, manipulation of the immune system was shown to attenuate the negative effects of inflammaging. For instance, surgical parabiosis of young and old mice can partially reverse deficits in neurogenesis, remyelination, and muscle regeneration, as mice share youthful plasma factors *via* the circulation (Ruckh et al., 2012; Katsimpardi et al., 2014; Sinha et al., 2014; Villeda et al., 2014). Similar effects were seen in bone marrow transplantation, which ameliorated age-related frailty and reduced hemorrhagic transformation after stroke (Heidt et al., 2014; Ritzel et al., 2018).

Inflammaging leads to epigenetic changes in cells via DNA methylation (De Martinis et al., 2005; Perez-de-Puig et al., 2015; Chen et al., 2016). In general, these genes are neurodegenerative and linked to exacerbated immune responses and cellular oxidative stress, which are also commonly observed in neurodegenerative disorders including Alzheimer’s disease (Lee et al., 2000; Blalock et al., 2003; Verbitsky et al., 2004; Fraser et al., 2005; Rowe et al., 2007; de Magalhães et al., 2009; Lipinski et al., 2010; Zeier et al., 2011; Yuan et al., 2012). Some inflammatory signatures include (1) differentially overexpressed inflammatory genes C1QA, C3, C1QB, CTSS, FCGR2B, IGJ, LYZ, MGST1, GSTA1, S100A4, S100A6, ANXA3, ANXA5, IL18, IL18BP LILRB3, IGJ, RAB27A, RAB32, PTPRC, SWAP70, SLC11AL, ST14, TEP1, TREM2, and TYROBP; and (2) underexpressed mitochondrial genes (ATP5G3, NDUFB11, UQCRQ, and UQCRFS1; de Magalhães et al., 2009; Wang et al., 2012; Ianov et al., 2016). Other pro-inflammatory biological processes (e.g., autophagy, oxidative stress) are elevated with age, including the overexpression of LYZ, CLU, MGST1, GSTA1, S100A4, S100A6, ANXA3, and ANXA5 and the underexpression of mitochondrial genes ATP5G3, NDUFB11, UQCRQ, and UQCRFS1 (de Magalhães et al., 2009). Additionally, the complement system genes, including C1QA, C1QB, C1QC, C1S, C3, C3AR1, C4α, C4β, C5, and C5AR1, are significantly upregulated with age (Cribbs et al., 2012). Additionally, with age, angiogenesis and neurogenesis that may contribute to recovery are diminished, as pro-angiogenic genes ANGPT2 and VEGFA are significantly downregulated (Guo et al., 2021). Genes related to synaptic transmission, axonal projection, dendrite growth, and neuroplasticity show age-related downregulation (Blalock et al., 2003; Lu et al., 2004; Verbitsky et al., 2004; Burger et al., 2007; Aenlle and Foster, 2010; Zeier et al., 2011; Berchtold et al., 2019; Androvic et al., 2020). These include genes related to axon functions (ANKS1B, CLSTN3, CBLN1, CHRM2, CHRNA5, CYP19A1, DOC2A, DNM3, GABRA4, GAD1, GRIP1, GRID1, GRID2, GRIK3, GRM7, GRM8, GLRB, LIN7B, MAGREE1, SCAMP1, PRKACA, SLC2A3, SV2B, SYT6, YWHAZ) and neurite growth (BACE1, DCC, DPYSL2, DPYSL5, DCTN2, GOT1, KLHL1, MAP2K4, KCNJ12, PGR, PTPRN2, and TACR3; Ianov et al., 2016). Neurons in the peri-infarct exhibit axonal sprouting associated with recovery after stroke and increase pro-inflammatory genes IGF1 and SPP1 activation (Li et al., 2010). Consistently, Berchtold et al. showed in the aged human hippocampus that anti-aging genes are highly enriched in neuron projection, cell junction, and axon (Berchtold et al., 2019).

Microglial are the principal cells driving the innate immune response in the brain and are also impacted by inflammaging. Differentially expressed genes include C4B, CCL4, CCL8, CLEC7A, CST7, CYBB, LGALS3, MMP12, and SPP1 (Holtman et al., 2015; Grabert et al., 2016; Androvic et al., 2020). These genes are in line with the transcriptional profile of microglia in neurodegenerative diseases (Mathys et al., 2017; Friedman et al., 2018; Hammond et al., 2019). For instance, in an Alzheimer’s disease mouse model, microglia significantly overexpressed complement components (C3, C4B, and CFB), chemokines (CCL12, CCL3, CCL4, and CXCL16), and immune-related genes (H2-D1, AXL, APOE, and LGALS3B; Mathys et al., 2017); APOE, SPP1, and LPL are specifically upregulated in microglia surrounding amyloid plaques (Keren-Shaul et al., 2017). CCL3 and CCL4 are upregulated in humans with multiple sclerosis (Omari et al., 2005; Szczuciński and Losy, 2007; Hammond et al., 2019). Many upregulated inflammaging genes are also the signatures of other immune cells, such as astrocytes (ANLN, C4B, GFAP, LYZ2, NEAT1, PCDHB6, PLIN4, and SERPINA3N; Boisvert et al., 2018; Clarke et al., 2018) and oligodendrocyte precursor cells (RAB37 and TNFAIP2; Spitzer et al., 2019; Androvic et al., 2020).

Transcriptional changes in peripheral blood neutrophils from aged Beagle dogs, mice, and humans are overwhelmingly related to chromatin and cell cycle (Zhang et al., 2004; Hall et al., 2010; Grassi et al., 2018; Tabula Muris Consortium et al., 2018; Kalucka et al., 2020; Lu et al., 2021). Chromatin compaction is essential to cellular functions as it represses genome transcription (Janssen et al., 2018). Lu et al. showed that 18 histone-encoding genes are significantly downregulated with aging in mouse bone marrow cells. The same study showed that neutrophils experience age-dependent changes in chromatin organization (Lu et al., 2021), with increased median neutrophil nucleosomal occupancy (Brinkmann et al., 2004; Papayannopoulos, 2018) and chromatin condensation (Lu et al., 2021; Figure 4). Since chromatin decompaction is a limiting step of NETosis, this suggests that aging can directly influence NETosis (Neubert et al., 2018; Sollberger et al., 2018). Transcriptional changes with aging, specifically in neutrophils, are complex due to exogenous and endogenous confounding factors, such as the increased number of medications taken by older individuals (Perez-de-Puig et al., 2015). Thus, the genomic link between inflammaging and neutrophils and strategies to reverse neutrophil immunosenescence in the elderly population requires further investigation (Simmons et al., 2021).

Despite changes in receptor expression with aging (decreased CXCR4 expression as described below), circulating neutrophil counts remain stable in individuals without co-morbidities (Chatta et al., 1994; Van Avondt et al., 2022). Their production is not diminished (Corberand et al., 1986; Perez-de-Puig et al., 2015), rather, their functions and signaling pathways are impaired (Hazeldine et al., 2014; Sapey et al., 2014; Tseng and Liu, 2014; Simmons et al., 2021). Neutrophil mobilization from bone marrow and proliferative response to G-CSF are significantly reduced with aging (Chatta et al., 1993, 1994). Neutrophils from older individuals release more neutrophil elastase and proteolytic enzymes, spontaneously produce NETs, ROS, and cytokines (e.g., TNF-α; Tseng et al., 2012; Hazeldine et al., 2014; Zhang et al., 2015), express high levels of extracellular matrix-degrading enzymes (e.g., MMP-9), and increase degranulation (Carter et al., 2013; Figure 4). Several autophagy-related pathways are upregulated in neutrophils during aging, consistent with the age-dependent proteostasis loss (López-Otín et al., 2013). Since control of autophagy is critical for neutrophil differentiation (Riffelmacher et al., 2017), regulation of NET formation (Park et al., 2017), and degranulation (Bhattacharya et al., 2015), this can not only damage the vasculature but also blunt local immune response (McLaughlin et al., 1986; Chua and Laurent, 2006; Gomez et al., 2007; Eskan et al., 2012; Nomellini et al., 2012; Wulfert et al., 2012).

During organismal aging, neutrophils have an increase in basal levels of activation of MAPK pathways and PI3K signaling (Fortin et al., 2006; De Maeyer et al., 2020). Excessive PI3K signaling impairs neutrophil chemotaxis and migration toward the insult (Fulop et al., 2004; Hinojosa et al., 2009; Chen et al., 2014; Hazeldine et al., 2014). Since PI3K directs PIP_3_ phosphorylation and accumulation to the edge of neutrophil, inducing the molecular cascade that recruits elements needed for neutrophil environmental sampling and propulsion, inhibiting PI3K can improve neutrophil mobilization in aged individuals. Furthermore, increased PI3K activity could impair PI3K/SHIP1/PTEN balance and causes neutrophil to be insensitive to inflammatory signals (Sapey et al., 2014). Serum levels of CXCL1/IL-8 are significantly higher in the circulation of aged patients and animals, causing increased respiratory burst, altered neutrophil trafficking, and degranulation (Bréchard et al., 2005; Wright et al., 2014; Figure 2). CCL5/RANTES concentrations significantly increase in old mice after stroke (Desai et al., 2010; Sieber et al., 2011, 2014). This is associated with neutrophil migratory defects, diminished neutrophil cytokine production (Kirkwood, 2005; Dalboni et al., 2013; Qian et al., 2014), reduced host phagocytic ability to control pathogen burdens (Butcher et al., 2001; Weiskopf et al., 2009; Domon et al., 2016, 2018; Bou Ghanem et al., 2017), and poor stroke outcome (Terao et al., 2008; Dénes et al., 2010; Dhungana et al., 2013).

Neutrophil apoptosis (Tortorella et al., 2006; Gasparoto et al., 2012) and clearance to bone marrow (Martin et al., 2003; Buckley et al., 2006; Casanova-Acebes et al., 2013) are also reduced with aging *via* GM-CSF, LPS, and IL-2 stimulation and activation (Fülöp et al., 1997). This leads to a persistent release of inflammatory signals that recruit more immune cells (Sendama, 2020), such as IFN-I signaling (Androvic et al., 2020), causing a continuous influx of neutrophils in blood (Danton and Dietrich, 2003). Aged animals also under-express CXCR4, CXCL12, and CD44 with increased CXCR2 expression, reducing neutrophil clearance via efferocytosis and enhancing their circulation in blood (Hart et al., 1997; Furze and Rankin, 2008; Figure 2). CXCR4 is known to be a CXCR2 antagonist, as CXCR2 induces natural aging phenotypic changes in mice (Martin et al., 2003; Eash et al., 2010; Adrover et al., 2019; Yan et al., 2021; Figure 2). This difference in neutrophil phenotype might explain why neutrophil depletion using anti-Ly6G rescue behavior deficits in MCAO aged animals but not young animals (Perez-de-Puig et al., 2015; Roy-O’Reilly et al., 2020). When given anti-Ly6G treatment after a 60-min MCAO, young mice (3 months) showed no functional benefits in corner testing and hang-wire testing; while aged animals (21–22 months) showed significantly improved recovery without changes in long-term mortality (Roy-O’Reilly et al., 2020). This is because anti-Ly6G treatment is less efficient for CXCR2^−^ neutrophils in all mice spleen, blood and lungs (Pollenus et al., 2019). Moreover, these unique patterns in behavioral rescues may be independent of the improvement in gross tissue damage observed with anti-Ly6G in previous studies, which has no correlation with age (Perez-de-Puig et al., 2015; El Amki et al., 2020; Erdener et al., 2021).

Neutrophil endothelial adhesion (Niwa et al., 1989; MacGregor and Shalit, 1990; Fortin et al., 2006, 200; Nogueira et al., 2018) and transepithelial migration (Bou Ghanem et al., 2017) are preserved with age. Neutrophils that have undergone diapedesis into inflamed tissues can exhibit retrograde motility and re-enter the vascular lumen. This is called reverse trans-endothelial cell migration (rTEM) and is mediated by elevated CXCL1 production by tissue-resident vascular and perivascular cells (Buckley et al., 2006; Mathias et al., 2006; Woodfin et al., 2011; Perez-de-Puig et al., 2015; Owen-Woods et al., 2020; Figure 4). Neutrophils that undergo rTEM have distinct surface phenotypes as to circulating and tissue-resident neutrophils which drive inflammation (Buckley et al., 2006; Woodfin et al., 2011). rTEM neutrophil express elevated ICAM-1 and CD54 expression and reduced CXCR1 expression (Buckley et al., 2006; Woodfin et al., 2011; Weisenburger-Lile et al., 2019). In aged tissues, a high level of CXCL1 expression is sustained by increased expression of ACKR1 at endothelial cell junctions, which facilitates CXCR2 downregulation. Since CXCR2 is responsible for transmigrating neutrophils, this causes impaired neutrophil motility and directs them back into circulation (Marki and Ley, 2020; Barkaway et al., 2021). Neutrophil excessive production of elastase in aged mice also cleaves the endothelial junctional adhesion molecule JAM-C and triggers rTEM (Woodfin et al., 2011; Colom et al., 2015; Girbl et al., 2018; Owen-Woods et al., 2020; Barkaway et al., 2021). Increased neutrophil elastase and decreased JAM-C have been shown to induce rTEM in mice (Jin et al., 2019; Kim Y. R. et al., 2020). rTEM neutrophils have longer survival, lower apoptosis rate, slower passage through the microvasculature, and higher ROS release (Buckley et al., 2006; Perez-de-Puig et al., 2015), suggesting that they may inflict systemic damage if reinfiltrated into remote organs (Ellett et al., 2015). rTEM neutrophils also have elevated integrins and high ICAM-1 phenotype, which may support rTEM neutrophil aggregation, degranulation, and ROS release within small blood vessels (Owen-Woods et al., 2020). With aging, CXCL1 and ACKR1 upregulation can bring rTEM neutrophils back into the circulation and entrap them in downstream organs, leading to multiorgan failure (Ellett et al., 2015). rTEM process has been reported recently in ischemia/reperfusion model mouse, rabbit models, and human patients (Clark et al., 1991; Colom et al., 2015; Cho et al., 2017; Weisenburger-Lile et al., 2019).

## Aging exacerbates neutrophil pathogenicity in ischemia

IS is a well-recognized disease of aging, with 75%–89% of strokes occurring in people over 65 years old, and the incidence of stroke doubles each decade after the age of 55 years (Chen et al., 2010). Along with a higher incidence, aged patients have higher mortality, suffer more severe deficits (as measured by the NIH and mRS scales), and recover more slowly (Kammersgaard et al., 2004; Marini et al., 2004; Perez-de-Puig et al., 2015; Scapini et al., 2016; Benjamin et al., 2018). Thus, understanding age-related molecular mechanisms of ischemic injury is crucial to translate preclinical findings to clinical interventions (Chen et al., 2010). In the past few decades, despite significant breakthroughs made by preclinical studies in animal stroke models, there still is a substantial translational roadblock for stroke treatments. This is because unlike patients in clinical trials, most preclinical studies used young animals with a homogeneous genetic background (Chen et al., 2010; Roy-O’Reilly and McCullough, 2018). Biological variables such as age, sex, and comorbidities that profoundly affect the clinical outcome are often unaccounted for (Bosetti et al., 2017; McBride and Zhang, 2017). The complex factors underlying worsened outcomes in older individuals remain poorly defined, particularly in females, who are often underrepresented in research studies (van der Worp et al., 2005; Sohrabji et al., 2017; Tsivgoulis et al., 2017; Carcel et al., 2019). Older animals also have higher mortality and more severe neurological deficits, making them more challenging in research studies (Rosen et al., 2005; DiNapoli et al., 2008).

An explanation for increased risk and worse stroke outcomes with aging is the coupling of chronic inflammation in the aged brain and ischemic injury, which amplifies neurodegeneration and tissue loss (Li et al., 2018; Tsai et al., 2019). Aged mice showed increased risks of hemorrhagic transformation and higher neurological deficits with stroke (Liu et al., 2009, 2010, 2012; Manwani et al., 2013; Ritzel et al., 2018; Roy-O’Reilly et al., 2020). Reports on age-related infarct size in the literature are mixed. Many showed that aged mice have significantly smaller infarct sizes (Liu et al., 2009; Manwani et al., 2014; Zhao S. et al., 2017; Korf et al., 2022), while the opposite result has also been recorded (Shen et al., 2019; Liberale et al., 2021). Recent data suggests that this difference might be because inflammatory insults that compromise BBB are independent of infarct sizes (Dénes et al., 2011). Furthermore, ischemic functional deficits seen in behavior testing might also happen independently from histological damage (shown in infarct size, edema formation, and hemorrhagic transformation; Liu et al., 2009). This partially explains why anti-Ly6G treatment reduces tissue damage and infarct volume in MCAO animals regardless of age (Harris et al., 2005; Villa et al., 2007; Kenne et al., 2012; Cuartero et al., 2013; Herz et al., 2015; Wang et al., 2019; El Amki et al., 2020; Kang et al., 2020; Erdener et al., 2021), while it only rescued behavior deficits in aged animals (Roy-O’Reilly et al., 2020). When aged mice received young bone marrow, they showed improved motor responses and fewer brain-infiltrating neutrophils post-ischemia. On the contrary, young mice that received aged bone marrow had worse behavioral outcomes and mortality. Removal of peripheral immune cells *via* splenectomy also decreases stroke-induced inflammation and injury in aged mice, thus, improving cognitive recovery (Chauhan et al., 2018; Tsai et al., 2019). Aged mice given a fecal transfer of young mice’s microbiome, a modulator of immune activation, produced improved outcomes (Zhang et al., 2015).

Age is a critical factor in studying neutrophil dynamics in stroke, since aging alters neutrophil functions and older brains are more susceptible to IS injury (Schulte-Herbrüggen et al., 2006; Manwani et al., 2013; Chen et al., 2014; Venna et al., 2014; Grønhøj et al., 2017; Ritzel et al., 2018). In preclinical studies, aged animals have a higher basal neutrophil proportion in the bone marrow and brain and greater admission NLR, which is associated with increased hemorrhagic risk after IS (Jickling et al., 2015; Maestrini et al., 2015; Perez-de-Puig et al., 2015). Aged brain-infiltrating neutrophils have heightened elevated ROS and MMP-9 production that degrades the extracellular matrix and BBB and has been connected to enlarged infarct volume (Montaner et al., 2003; Tang et al., 2006; Perez-de-Puig et al., 2015). Aged neutrophils in the ischemic brain also have defective debris clearance, leading to more risk of tissue death (Chen and Sun, 2007; Arumugam et al., 2010). Old animals have higher levels of circulating CXCL12 (increased CXCR4 expression) in the sham group but significantly lower levels of CXCL12 than young animals after IS, implying defects in neutrophil clearance (Roy-O’Reilly et al., 2020). Following IS, aged mice were also shown to have elevated neutrophil-activating IL-6 and CXCL1 compared to young animals (Roy-O’Reilly et al., 2020). Depletion of neutrophils via a specific monoclonal antibody after IS led to long-term benefits in functional outcomes only in aged animals. These results demonstrate that aging is tightly linked to neutrophil pathogenicity in IS, and that neutrophil-targeted therapies may confer greater benefit in aged subjects than the young ones (Perez-de-Puig et al., 2015; Figure 3).

In clinical studies, it has been shown that circulating neutrophils in IS patients are hyperactive and can produce significantly more ROS both in non-inflammatory and inflammatory conditions than the controls. These neutrophils bear a lower CD62L expression and higher CD11b expression, suggesting that they are mature neutrophils and can readily and rapidly respond to inflammatory stimuli. Patients with IS also have lower levels of circulating NETosis products than the controls, as well as higher circulating levels of JAM. Serum neutrophil elastase levels are also higher in IS patients (Weisenburger-Lile et al., 2019).

Previous gene expression studies of experimental stroke using microarrays (Jin et al., 2001; Schmidt-Kastner et al., 2002; Kim et al., 2004; Ford et al., 2006; Mitsios et al., 2007; Jeyaseelan et al., 2008; Hori et al., 2012) and more recently RNA sequencing (Dergunova et al., 2018; Androvic et al., 2020) have provided useful insights into the pathophysiology of IS. However, few studies included aged animals or focused on neutrophils. Androvic et al. found in aged post-ischemic mice an upregulation of more than 400 genes, many involved in the inflammatory cascade, and accompanied by a great influx of neutrophils (Androvic et al., 2020). Gene ontology terms related to inflammatory responses (e.g., IFN-1 signaling) and cellular organization are overexpressed in aged MCAO mice. Consistent with findings on inflammaging transcriptional changes, synaptic communication and plasticity-related genes are downregulated more in the aged MCAO group (Blalock et al., 2003; Lu et al., 2004; Verbitsky et al., 2004; Burger et al., 2007; Aenlle and Foster, 2010; Zeier et al., 2011; Berchtold et al., 2019; Androvic et al., 2020). These include CADPS, PNKD, LIN7A, BRAF, DNM1, RIMS1, CPLX1, SYT2, and NRXN3, indicative of impaired synaptic transmission (Androvic et al., 2020). Strikingly, chemotaxis and leukocyte migration are among the most enriched gene categories in these animals, which aligns with reports on neutrophil infiltration and poor stroke outcomes in the aged (Engler et al., 1983; del Zoppo et al., 1991; Pham and Bendszus, 2016; Androvic et al., 2020; El Amki et al., 2020; Table 1). Pathways associated with cytokine secretion, oxidative phosphorylation, and cell cycle regulation (e.g., ERK/MAPK signaling, cAMP/cGMP-mediated signaling) are significantly enriched, suggesting an inflated neuroinflammatory environment contributing to secondary damage in aged MCAO mice (Androvic et al., 2020). The persistent upregulation of pro-inflammatory (CXCL12, MMP8, MMP12, MMP14, MPEG1, TNFRSF1a, and TNFRSF1B) and genes causing fibrotic scar buildup (CTHRC1, IL6RA, IL13AR1, IL18, MMP2, RASSF4, TGFB1, TGFB2, and TIMP-1) diminish angiogenesis response in aged MCAO rats. The upregulation of pro-angiogenic genes (ANGPT2, ANGPTL4, CIB1, COL8A1, NRP1, PECAM1, LEF1, PTTG1IP, RAC2, RUNX1, TNP4, and WNT4) are delayed in aged MCAO rats, as well as and basal lamina/extracellular matrix reconstruction genes (COL4A2, FN1, LAMC1, NID2, PLOD3), compared to the young ones (Buga et al., 2014; Table 1). Preclinical studies of aged individuals are essential as the behavior of neutrophils in young and aged individuals may not be the same, and as almost all stroke patients are old, it is essential to study neutrophil dynamic changes with aging.

**Table 1:** Age-dependent transcriptional profile post-ischemia.

Functions	Differentially expressed genes	Activity	Reference
Mitochondria functions	ATP5G3, NDUFB11, UQCRQ, UQCRFS1	Upregulation	Androvic et al. (2020)
Astrocyte functions	ANLN, C4B, GFAP, LYZ2, NEAT1, PCDHB6, PLIN4, SERPINA3N	Upregulation	Androvic et al. (2020)
Oligodendrocyte precursor cells functions	RAB37, TNFAIP2	Upregulation	Androvic et al. (2020)
Microglia functions	C4B, CCL8, CLEC7A, CST7, CYBB, LGALS3, MMP12, SPP1	Upregulation	Androvic et al. (2020)
Angiogenesis	ANGPT2, VEGFA ANXA1, ADAMTS9, CEACAM1, GATA2, ADGRA2, ADGRG3, ANKRD1	Downregulation Upregulation	Guo et al. (2021) and Androvic et al. (2020)
Inflammasome	AIM2	Upregulation	Kim H. et al. (2020)
Chemotaxis, leukocyte migration	BST1, CXCL1, CXCL9, CXCL11, CXCL13, CXCR2, DRAXIN, LGMN, TREM1	Upregulation	Androvic et al. (2020)
Regulation of immune response	ARG1, IL7R, FCGGR2B, NOD2, SH2D1B1, CEACAM1, CD274	Upregulation	Androvic et al. (2020)
cAMP/cGMP signaling	MRAP, PTGER2, NPR1, HTR2B	Upregulation	Androvic et al. (2020)
Cytokine secretion	GGSDMD, TRIM16, DDX58, IFIH1, CLEC5A, NOD2	Upregulation	Androvic et al. (2020)
Cytokine/chemokine receptor binding	CCR1, CCR5, CXCL9, CXCL11, CXCL13, CXCR2, CXCR6, OSM, PF4, LEPR	Upregulation	Androvic et al. (2020)
Type 1 interferon signaling	STAT1, LRF9, RSAD2, MX2, OAS3, USP8, DDX58, ISG15, LFI203, LFI204, LFIT1, LFIT2, LFIT3, GBP2, GBP3	Upregulation	Androvic et al. (2020)
Synaptic communication	CADPS, PNKD, LIN7A, BRAF, DNM1, RIMS1, CPLX1, SYT2, NRXN3	Downregulation	Blalock et al. (2003), Lu et al. (2004), Verbitsky et al. (2004), Burger et al. (2007), Aenlle and Foster (2010), Zeier et al. (2011), Berchtold et al. (2019), and Androvic et al. (2020)
Fibrotic scar buildup	CTHRC1, IL6RA, IL13AR1, IL18, MMP2, RASSF4, TGFB1, TGFB2, TIMP-1	Upregulation	Buga et al. (2014)

Ischemic stroke in aging individuals prompts distinct transcriptional changes compared to young individuals. Differentially expressed genes corresponding to cellular and biological functions, as well as their activity are compiled from different studies.

## Potential therapeutic targets and future research directions

Numerous studies over the years have shown that aging exacerbates stroke pathology in both human patients and experimental models (Li et al., 2005, 2005; Ritzel et al., 2016). Therefore, investigating the molecular mechanisms that underlie the effects of age on stroke, especially in light of the aging population, is important (Camici and Liberale, 2017). Inflammaging, or more specifically, age-related changes in neutrophils, could constitute a novel therapeutic target. Many studies have shown that blocking neutrophil-associated pathways, such as reactive species production, can reduce infarct volume (Jickling et al., 2015). However, clinical translation of these results still faces many challenges (Mitsios et al., 2007; Warner et al., 2014; Yang and Paschen, 2017). Experiments in aged animals are needed for better translation to clinical trials (Manwani et al., 2013).

Aging and IS involve similar pathophysiology, particularly heightened inflammation (Spescha et al., 2013, 2015; Camici et al., 2015; Diaz-Cañestro et al., 2019; Liberale et al., 2020). To achieve the maximum protective effect, further experiments must be conducted to determine whether promising anti-inflammatory treatments targeting neutrophils should be administered well before the initiation of recanalization therapy (Kollikowski et al., 2022). It is necessary to include aged animals in both mechanistic and therapeutic studies examining neutrophil contributions to acute IS since previous anti-neutrophil therapies have only been tested on young animals (Benjamin et al., 2019).

## Author contributions

TAB researched and wrote the manuscript and created the figures. IW and GJ co-wrote and edited the manuscript and figures. All authors contributed to the article and approved the submitted version.

## Funding

This work was supported by CIHR (180224) and HSFC (G-19-0026316) funding to IW.

## Conflict of interest

The authors declare that the research was conducted in the absence of any commercial or financial relationships that could be construed as a potential conflict of interest.

## Publisher’s note

All claims expressed in this article are solely those of the authors and do not necessarily represent those of their affiliated organizations, or those of the publisher, the editors and the reviewers. Any product that may be evaluated in this article, or claim that may be made by its manufacturer, is not guaranteed or endorsed by the publisher.
